# Public Knowledge, Attitudes, and Practices Towards Antibiotic Use and Antimicrobial Resistance in the Western Region of Saudi Arabia

**DOI:** 10.7759/cureus.31857

**Published:** 2022-11-24

**Authors:** Mokhtar Shatla, Fadi S Althobaiti, Abdulaziz Almqaiti

**Affiliations:** 1 Community Medicine and Pilgrims Health Care, Umm Al-Qura University, Makkah, SAU; 2 Family Medicine, Menoufia University, Menoufia, EGY; 3 Medicine, Umm Al-Qura University, Makkah, SAU

**Keywords:** antibiotics practices, western region, saudi arabia, antibiotics attitudes, antibiotics knowledge, antibiotics awareness, antibiotics resistance, antimicrobial resistance, antibiotics

## Abstract

Introduction

Antimicrobial resistance (AMR) is one of the major concerns for global health. Inappropriate use of antibiotics is speeding up the process of AMR. The aim of this study was to assess the knowledge, attitude, and practices (KAP) of the population of the western region of Saudi Arabia towards antibiotic use and AMR.

Methods

A cross-sectional study was conducted using an online questionnaire via social media platforms. It consisted of four parts: first, the participant's sociodemographic characteristics. Second, an assessment of the knowledge of antibiotic use and AMR. The third and fourth parts are to evaluate the attitude and practices towards antibiotic use and AMR.

Results

From a total of 501 participants, 379 (75.6%) were females, and 354 (70.7%) of the participants had a college degree. Regarding the overall knowledge of antibiotics and AMR, 277 (55.29%) of the respondents had poor knowledge. In addition, 443 (88.42%) of the participants also reported having an inadequate attitude. Moreover, inadequate practices related to the use of antibiotics were also reported among 305 (60.89%) of the participants. Factors associated with the level of knowledge are gender, age, educational level, and occupational status. Regarding occupation, being in a medical job was associated with a better knowledge of antibiotics and AMR than other occupations.

Conclusion

Our study revealed poor overall knowledge, attitudes, and practices toward antibiotics and AMR among participants. Conducting educational health campaigns for the public that aim to promote awareness and enhance good practices, emphasizing the role of health care providers in health education for the public, and enforcing strict regulations to control the non-prescription dispensing of antibiotics will help to combat this issue. However, future research on this topic is required.

## Introduction

Antimicrobials, including antibiotics, antivirals, antifungals, and antiparasitics, are used to prevent and treat infections in humans, animals, and plants [1]. Antimicrobial resistance (AMR) occurs when microbes change over time, making antimicrobials ineffective [1]. AMR can occur in microbes naturally, but it can also be induced by inappropriate use of antimicrobials or unintentional exposure [2]. This results in organisms that are very challenging to treat and have a significantly high fatality rate [1].

AMR is spreading, creating microbes that cannot be treated with existing medications. Currently, common organisms that cause different diseases, such as tuberculosis (TB), HIV/AIDS, malaria, sexually transmitted diseases, urinary tract infections, lung infections, and blood infections, can resist a wide variety of antimicrobics [2]. Two prior studies conducted in Jeddah, Saudi Arabia, showed a high prevalence of HIV and TB resistance. Regarding HIV, 41%, 16%, and 13% were resistant to nucleoside reverse transcriptase inhibitors (NRTIs), non-nucleoside reverse transcriptase inhibitors (NNRTIs), and protease inhibitors (PI), respectively. While in TB, among 901 cases, drug-resistant TB was observed in 193 (21.4%) [3-4].

AMR is significantly costly, not just financially but also in terms of global health, food security, environmental well-being, and socio-economic development. At present, AMR causes approximately 700,000 deaths worldwide each year [2]. In addition, there is approximately one death every 10 minutes in Europe or the United States of America because of deadly infections caused by antimicrobial-resistant bacteria [5]. AMR is rising in almost every country [5]. However, a systemic literature review in Saudi Arabia demonstrated that, in comparison to the 1990s, there has been a significant increase in carbapenem-resistant gram-negative bacilli (GNB) over the last decade. Additionally, it also revealed an increase in the prevalence of extended-spectrum beta-lactamase-producing bacteria in Saudi Arabia, with 29% documented among *Escherichia coli* (*E. coli*) and 65% among *Klebsiella pneumoniae* (*K. pneumoniae*). Accordingly, these documented rates were associated with multiple reported outbreaks, and mortalities ranged between 11% and 40% [6].

A study in 2015 from Riyadh showed a dramatic reduction in the susceptibilities of *Acinetobacter baumannii* (*A. baumannii*) to meropenem and imipenem, where the susceptibilities were 64-81.2% in 2006 and 8.3-11% in 2012. Consequently, with carbapenem resistance, therapeutic options are extremely limited, making it a major epidemiological concern [7]. In Saudi Arabia in 2014, a national surveillance of AMR among gram-positive cocci represented a high resistance rate among *Staphylococcus aureus* (*S. aureus*), 32% were methicillin-resistant (MRSA), and among* Streptococcus pneumoniae*, 33% were resistant to penicillin G, and 26% were resistant to erythromycin [8].

Regarding multi-drug resistant bacteria in Saudi Arabia, there are many factors associated with its increase. It is well-established that inappropriate use of antibiotics is an important factor in the development of AMR [6]. Over-the-counter antibiotics without a prescription is another factor leading to the inappropriate use of antibiotics, which contributes to more antimicrobial resistance.

A study in 2001 was done in the eastern province of Saudi Arabia and revealed that only one pharmacy out of 88 refused to dispense medication without a prescription for patients claiming to have a UTI [9]. Another study in 2011 was done in Riyadh among 327 pharmacies and showed that 77.6% of pharmacies dispensed antibiotics without a prescription [10]. Lack of awareness is another major factor in the misuse of antibiotics worldwide [11]. Patients' knowledge, attitude, and practice (KAP) are important factors contributing to the misuse of antibiotics [11-12].

A study in the Hail region of 500 participants found that 26% got antibiotics without a prescription, only 34% finished the full antibiotic course, and 36.2% were unaware of antibiotic resistance and its rise [12]. In addition, a recent study in 2021 to assess KAP towards antibiotic use and AMR among the general public in Saudi Arabia showed an insufficient attitude toward antibiotic use. It also suggests reducing antibiotic misuse by raising awareness [13]. Low knowledge levels were also reported in a 2021 study in Jeddah, indicating the need to increase public knowledge and raise awareness [14]. Globally, in Kuwait, a KAP study on antibiotic use in 2015 [15] reported that 72.8% of the respondents had received an antibiotic prescription in the year before the survey; of those, 36% did not complete the course of treatment. Moreover, 27.5% were self-medicating primarily for cough, sore throat, and a common cold. A 2021 study conducted in Boyolali, Indonesia, revealed that most of the participants had insufficient knowledge about the proper usage, accessibility, and function of antibiotics. Male sex, low income, low educational levels, and residency in rural areas were associated with inappropriate use of antibiotics [16].

To implement corrective measures regarding antibiotics KAP, first, we need to know the level of awareness in society currently. Therefore, the aim of this study was to assess the knowledge, attitude, and practices (KAP) of the population of the western region of Saudi Arabia towards antibiotic use and antimicrobial resistance.

## Materials and methods

Study design

The study design was cross-sectional and conducted using a validated questionnaire that had been developed and used in a previous study [10]. The survey was conducted during the period from June to July 2022. This survey aims to evaluate public knowledge, attitudes, and practices among people living in the western region of Saudi Arabia towards antibiotic use and antimicrobial resistance.

Study population

The study was conducted via an online questionnaire, and our target sample in this study was public adults who are Arabic and English-speaking individuals living in the Western Region of Saudi Arabia. This study excluded non-Arabic, non-English-speaking individuals and individuals living outside the Western Region of Saudi Arabia.

Sampling methodology

The sample was conducted on public people from the 1st of June to the 1st of July 2022 in the Western Region of Saudi Arabia. We obtained consent from the participants before they started filling out the questionnaire. According to OpenEpi version 3.1, the sample size should not be less than 769 participants. However, the final sample size that has been included in this study is 501. The questionnaire form is online, and it has been sent through social media platforms. It consists of four parts of close-ended type questions in the form of multiple-choice answers. It was made in Arabic and English languages. The first part included eight items of the participant's sociodemographic characteristics (gender, age, place of living, marital status, education level, occupational status, monthly income, and source of information). The second part is made up of 17 items to assess the knowledge of antibiotic use and antimicrobial agents. The third part has ten items that evaluate the attitude towards antibiotic use and AMR using a five-point Likert scale (strongly agree, agree, uncertain, disagree, strongly disagree). The fourth part is about assessing the participant's practice towards antibiotic use and AMR, and it is composed of 17 questions using a five-point Likert scale (never, rarely, sometimes, often, always). Data was collected from any participant who met our criteria. The electronic data collection forms did not show any nominative information. Data was automatically entered into the Excel sheet (Microsoft, Redmond, Washington). After verification, we transferred this data to the SPSS software (IBM Inc., Armonk, New York) for analysis.

Data analysis

After the data was extracted, it was revised, coded, and fed to the statistical software SPSS version 25. The results were reported as frequency and percentages. A scoring system was adopted based on Awad and Aboud, 2015 [15]. Descriptive statistics were obtained for sociodemographic variables. The knowledge, attitudes, and practices (KAP) scores were calculated as continuous variables by summing the number of correct responses to the total statements for each category (KAP). One point was assigned to each correct response and zero otherwise. Eighty percent and above were assigned as a good score for each category (KAP), and less than 80% were identified as having a poor score. Descriptive analysis based on frequency and percent distribution was done for all variables, including participants' age, gender, education level, marital status, occupation, and income. Also, participants' knowledge, attitude, and practice items were tabulated and graphed. Cross-tabulation was used to assess factors associated with the knowledge level regarding antibiotics and antimicrobial resistance. The Chi-squared test of independence is used for testing associations.

Ethical part and confidentiality

Consent was taken from each participant in the questionnaire. The objectives of the research were explained to the study's participants. Additionally, their consent was obtained prior to participating. They were also informed that participation was entirely voluntary, and that no personal information would be requested of them. All participants' identities were kept anonymous and confidential. Responses were accessed by the research investigators only. Ethical approval was sought from the Biomedical Ethics Committee of Umm Al-Qura University (UQU).

## Results

A total of 629 participants completed the study questionnaire. One hundred twenty-eight were excluded as they were not from the western region, and 501 participants were included as they fulfilled our inclusion criteria. Table 1 shows the demographic characteristics of the sample participants (n=501), females (n=379, 75.6%), and males (n=122, 24.4%). Of the total respondents, 255 (50.9%) were in the age group of 18-24 years. Moreover, the majority (354, 70.7%) had a college degree, 230 (45.9%) were students, and 303 (60.5%) of the study population earned a monthly income of less than 3000 SAR.

**Table 1:** Sociodemographic characteristics (n=501)

Variable	Response options	n	%
Gender	Male	122	24.4
	Female	379	75.6
Age	< 18	11	2.2
	18-24	255	50.9
	25-34	91	18.2
	35-44	87	17.4
	> 44	57	11.4
Marital status	Single	303	60.5
	Married	184	36.7
	Divorced	11	2.2
	Widowed	3	0.6
Education level	Primary or below	9	1.8
	Middle school	11	2.2
	High school	104	20.8
	College	354	70.7
	Masters or PhD	23	4.6
Occupational status	Student	230	45.9
	Unemployed	136	27.1
	Medical job	22	4.4
	Non-medical job	113	22.6
Income (monthly)	< 3000	303	60.5
	3000-4999	43	8.6
	5000-10,000	89	17.8
	>10,000	66	13.2

Table 2 shows the percentage of responses to each of the seventeen knowledge questions related to antibiotic use. Of the total respondents, 85.4% knew that antibiotics are not considered over-the-counter drugs, 86.2% knew that they can be used to treat a bacterial infection, 95.8% knew that some patients may have an allergy to specific antibiotics, 73.9% knew that not completing the full course of antibiotics may cause antibiotic resistance, 83.4% knew that antibiotic might kill the beneficial bacteria in skin, stomach or intestine, and 94.2% were aware that overuse of antibiotics could cause resistance to other antibiotics.

**Table 2:** Respondents' knowledge about antibiotics use (n=501)

Knowledge questions related to antibiotic use	No		Yes		Correct option	
	n	%	n	%	n	%
Antibiotics are considered over-the-counter drugs	428	85.4%	73	14.6%	428	85.4%
Antibiotics are medicines used to treat bacterial infections	69	13.8%	432	86.2%	432	86.2%
Antibiotics are medicines used to treat viral infections	245	48.9%	256	51.1%	245	48.9%
Antibiotics are medicines used to treat a cold and cough	253	50.5%	248	49.5%	253	50.5%
Antibiotics are medicines used to treat any illness with a fever	293	58.5%	208	41.5%	293	58.5%
Some patients may have an allergy to specific antibiotics	21	4.2%	480	95.8%	480	95.8%
Some antibiotics can cause diarrhea	68	13.6%	433	86.4%	433	86.4%
Overuse of antibiotics could cause resistance to other antibiotics	29	5.8%	472	94.2%	472	94.2%
Antibiotics may cause drug interaction and reduce certain drugs efficacy	53	10.6%	448	89.4%	448	89.4%
Antibiotics may kill our beneficial bacteria in the skin, stomach, or intestines	83	16.6%	418	83.4%	418	83.4%
Antibiotics do not cause side effects	440	87.8%	61	12.2%	440	87.8%
Using antibiotics when they are not necessary leads to antibiotic resistance	60	12.0%	441	88.0%	441	88.0%
Not completing the full course of antibiotics may cause antibiotic resistance	131	26.1%	370	73.9%	370	73.9%
Using antibiotics without a physician's prescription has nothing to do with antibiotic resistance	304	60.7%	197	39.3%	304	60.7%
Bacteria that normally live on the skin and in the gut are harmful to your health	373	74.5%	128	25.5%	373	74.5%
Missed doses should be taken as soon as you remember	148	29.5%	353	70.5%	353	70.5%
A missed dose should be skipped	290	57.9%	211	42.1%	290	57.9

**Figure 1:**
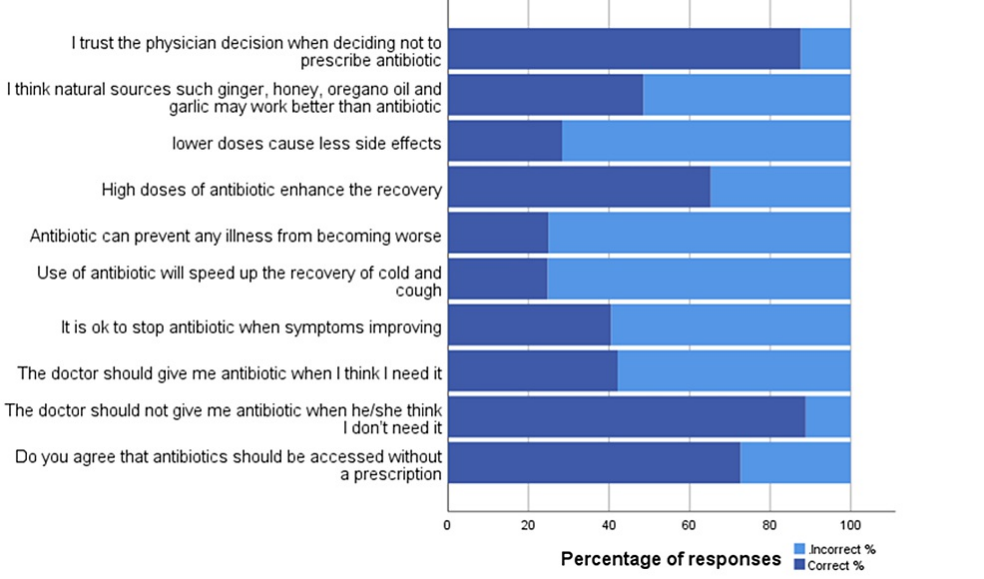
Respondents' attitude towards antibiotics use (n=501)

Figure 1 shows the percentage of responses to each of the ten attitude questions related to antibiotic use. Of the total respondents, 87.6% trusted the physician's decision when deciding not to prescribe antibiotics, and the majority (88.9%) believed that doctors should not prescribe antibiotics when not needed. Similarly, 72.6% did not agree that antibiotics should be accessed without a prescription. On the other hand, 40.5% did not agree to stop antibiotics when symptoms improved. Moreover, 24.8% disagree the use of antibiotics will speed up the recovery from a cold and cough.

**Figure 2:**
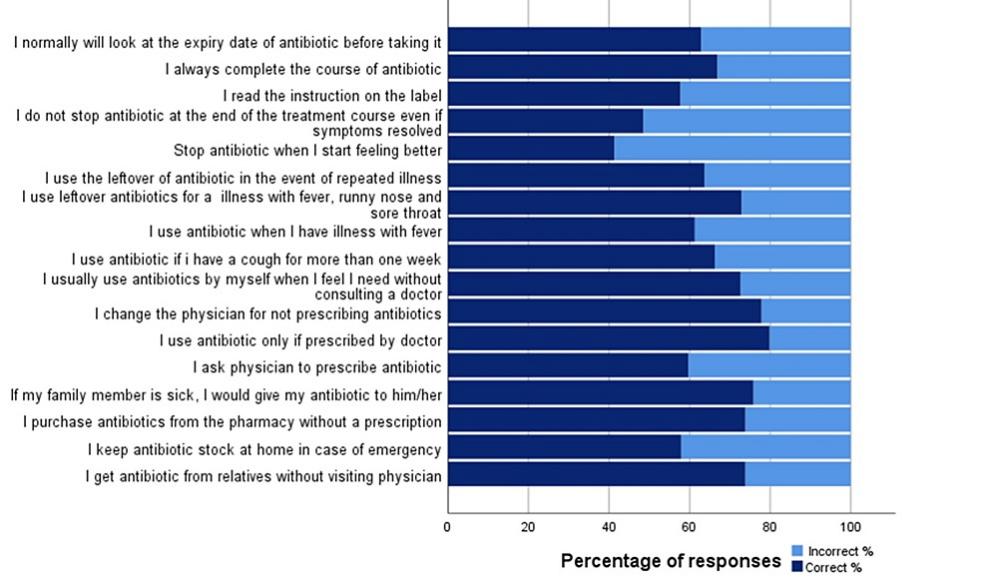
Respondents' practices towards antibiotics use (n=501)

Figure 2 shows the percentage of responses to each of the seventeen practice questions related to antibiotic use. Of the total respondents, 73.9% did not get antibiotics from relatives without visiting a physician, and 57.9% did not keep antibiotic stock at home in case of emergency. Moreover, the majority (73.9%) disagree to purchase antibiotics from the pharmacy without a prescription, and 77.9% disagree to change the physician for not prescribing antibiotics. However, 57.7% believe in reading the instruction on the label.

**Figure 3:**
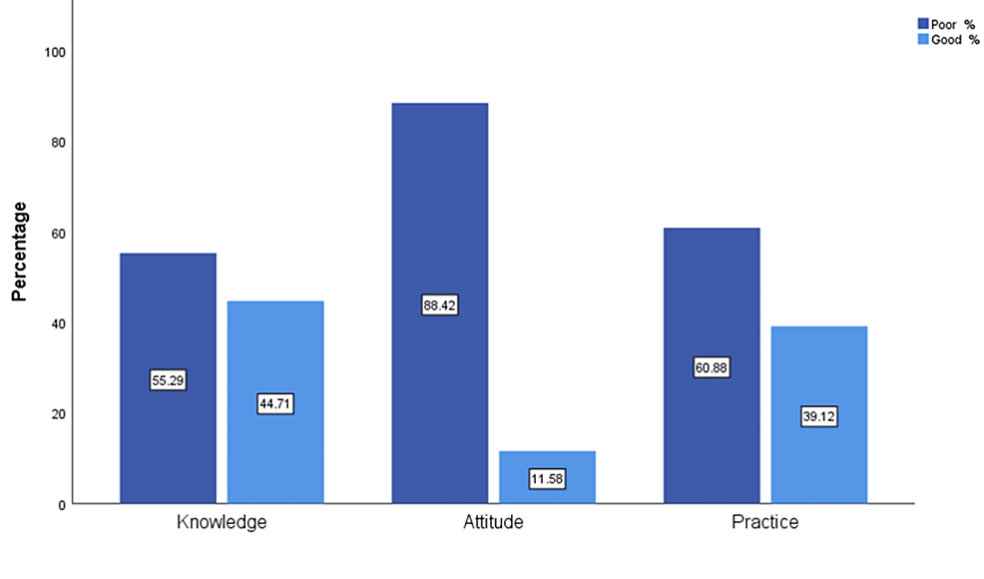
Overall KAP score levels toward antibiotics use KAP - knowledge, attitude, and practices

Figure 3 shows that most of the respondents had poor knowledge (277, 55.29 %), attitude (443, 88.42%), and practice (305, 60.88%) related to the use of antibiotics. The mean ± standard deviation of the knowledge score was 12.92 ± 2.61, attitude was 5.235 ± 1.99, and practice was 11.12 ± 4.37.

Table 3 shows an association between knowledge of antibiotics use and social-demographic variables. Knowledge and sociodemographic variables are categorical variables; therefore, the Chi-square test of independence is used for testing the association between them. Significant factors associated with knowledge were gender, age, educational level, and occupational status (p<0.05). In contrast, marital status and income have no significant association with participants' knowledge. Among significant factors, only those participants from medical jobs had better knowledge than respondents' from other occupations.

**Table 3:** Association between knowledge of antibiotics and sociodemographic variables *p<0.05 **p<0.01 poor/good within demographic variables + Cell frequency is less than 5, Fisher's exact test is used.

Variables	Response options	Poor (%)	Good (%)	χ2	P
Gender	Male	81 (66.4)	41 (33.6)	8.044	0.005^**^
	Female	196 (51.7)	183 (48.3)		
Age	< 18	11 (100)	0 (0)	12.630^+^	0.012^*^
	18-24	131 (51.4)	124 (48.6)		
	25-34	52 (57.1)	39 (42.9)		
	35-44	52 (59.8)	35 (40.2)		
	> 44	31 (54.4)	26 (45.6)		
Marital status	Single	166 (54.8)	137 (45.2)	2.561^+^	0.468
	Married	103 (56)	81 (44)		
	Divorced	5 (45.5)	6 (54.5)		
	Widowed	3 (100)	0 (0)		
Education level	Primary or below	4 (44.4)	5 (55.6)	11.841^+^	0.016^*^
	Middle school	10 (90.9)	1 (9.1)		
	High school	62 (59.6)	42 (40.4)		
	College	184 (52)	170 (48)		
	Masters or PhD	17 (73.9)	6 (26.1)		
Occupational status	Student	113 (49.1)	117 (50.9)	12.178	0.007^**^
	Unemployed	85 (62.5)	51 (37.5)		
	Medical job	8 (36.4)	14 (63.6)		
	Non-medical job	71 (62.8)	42 (37.2)		
Income (monthly)	< 3000	160 (52.8)	143 (47.2)	4.855	0.183
	3000-4999	30 (69.8)	13 (30.2)		
	5000-10,000	48 (53.9)	41 (46.1)		
	>10,000	39 (59.1)	27 (40.9)		

**Figure 4:**
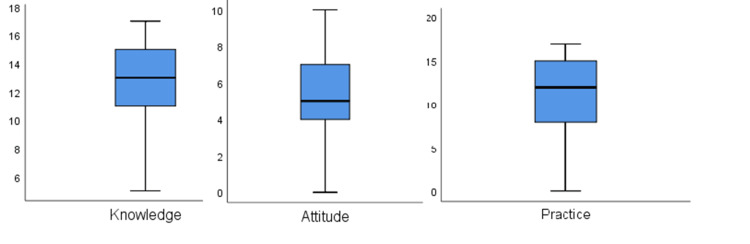
Boxplot of knowledge, attitude, and practices

Differences in the mean score of attitude and practice towards antibiotics use related to gender were tested using the Mann-Whitney U test, which is an alternative to the independent sample t-test in case the normality assumption is not fulfilled. For other variables, nonparametric alternative to ANOVA (Kruskal-Wallis) test is used [16]. Kolmogorov-Smirnov and Shapiro-Wilk normality test results are shown in Table 4. Since p<0.05 for all the variables, which concludes that the normality assumption, is not fulfilled. Similarly, a boxplot also confirms the non-normality of knowledge, attitude, and practice score (Figure 4). Therefore, nonparametric alternatives to t-test and ANOVA are suitable for the analysis.

**Table 4:** Normality test for knowledge, attitude, and practices

	Kolmogorov-Smirnov			Shapiro-Wilk		
	Statistic	df	Sig.	Statistic	df	Sig.
Knowledge	.114	501	.000	.960	501	.000
Attitude	.116	501	.000	.973	501	.000
Practice	.153	501	.000	.915	501	.000

The mean ± standard deviation of attitude was 5.235 ± 1.99. This score was significantly (p<0.05) lower in men (4.5983 ±2.1190) than in women (5.4406±1.9144) (Table 5). The mean ± standard deviation of practice was 11.12 ± 4.37. This score was significantly (p<0.05) lower in men (8.7869 ± 0.42280) than in women (11.8813± 0.20447) (Table 5). Another significant factor of attitude and practice is age. Marital status is only significant for practice. Furthermore, the mean scores of education level, occupational status, and income were insignificant for both attitude and practice.

**Table 5:** Differences in the mean score of attitude and practice towards antibiotic use related to demographic variables * p<0.05 ** p<0.01 *** p<0.001

Variables	Response options	Attitude		Practice	
		Mean ± SD	p-value	Mean ±SD	p-value
Gender	Male	4.5983 ± 2.1190	0.000^***^	8.7869 ± 0.42280	0.000^***^
	Female	5.4406± 1.9144		11.8813± 0.20447	
Age	< 18	3.6363± 2.5009	0.015^**^	8.9091 ± 5.1274	0.028^*^
	18-24	5.0509± 1.9545		10.6941± 4.4580	
	25-34	5.4175± 2.0443		12.0694± 4.3507	
	35-44	5.5517± 1.9869		11.5747± 4.0192	
	> 44	5.5964± 1.8406		11.1277± 4.3767	
Marital status	Single	5.0330 ± 2.0227	0.054	10.7162 ± 4.4734	0.012^*^
	Married	5.5489 ± 1.9579		11.7663 ± 4.2162	
	Divorced	5.5454 ± 1.5075		13.000 ± 1.9493	
	Widow	5.3333 ± 1.1547		6.6667 ± 3.0550	
Education level	Primary or below	5.2222 ± 2.4888	0.234	10.333 ± 5.0497	0.374
	Middle school	3.6363 ± 2.3779		8.7273 ± 5.7635	
	High school	5.1250 ± 2.0322		10.855 ± 4.2664	
	College	5.3107 ± 1.9540		11.336 ± 4.3510	
	Masters or PhD	5.3478 ± 1.9448		10.608 ± 4.1859	
Occupational status	Student	5.1217 ± 1.9918	0.262	11.000 ± 4.2713	0.666
	Unemployed	5.2205 ± 1.9462		11.404 ± 4.4958	
	Medical job	6.0454 ± 1.9634		11.181 ± 4.6356	
	Non-medical job	5.3274 ± 2.0591		11.044 ± 4.4368	
Income (monthly)	< 3000	5.1518 ± 1.9283	0.053	11.347 ± 4.3339	0.247
	3000-4999	5.0000 ± 2.0931		10.279 ± 4.0492	
	5000-10,000	5.1461 ± 2.1243		10.752 ± 4.7536	
	>10,000	5.8939 ± 1.9855		11.636 ± 4.2304	

Spearman's correlation was used to check the relationship between KAP. There is a significant relationship between the three variables (p<0.05) (Table 6). The highest correlation was found between attitude and practice (0.533), followed by 0.525 for the relationship between attitude and knowledge, and then 0.505 for the relationship between knowledge and practice.

**Table 6:** Correlation between knowledge, attitude, and practices of respondents towards antibiotics use *** p<0.001

	Knowledge	Attitude	Practice
Knowledge	1	0.525^***^ (0.000)	0.505^***^ (0.000)
Attitude	0.525^***^ (0.000)	1	0.533^*** ^(0.000)
Practice	0.505^***^ (0.000)	0.533^***^ (0.000)	1

## Discussion

This is a cross-sectional study to assess KAP towards antibiotics and AMR in the western region of Saudi Arabia. A validated questionnaire from a literature review [13] was used to collect the data. Our study showed insufficient knowledge, an inadequate attitude, and incorrect practices related to the use of antibiotics.

Among the most important findings in the knowledge part, a significant percentage of respondents had low knowledge regarding the role of antibiotics. Almost half (51.1%) of the respondents think that antibiotics can be used to treat viral infections, while 49.5% think they can be used for a cold and cough. Moreover, 41.5% agreed that antibiotics are medications used to treat any illness with fever. However, most of the respondents (88.0%) knew that unnecessary use of antibiotics leads to antibiotic resistance, and the majority (85.4%) knew that antibiotics are not considered over-the-counter drugs. This is in comparison to a previous study that was done in 2021 across all five regions of Saudi Arabia [13], which shows that 76.5% knew that antibiotics are used in cases of bacterial infection, and 74.9% knew that antibiotic resistance may be caused by not completing the course of the antibiotic. In addition, 67.5% knew that the beneficial bacteria that normally live on the skin or in the stomach/intestines might be killed by antibiotics, and only 56.9% knew that antibiotics should not be used to treat viral infections. The same study shows that 88% of the respondents had good knowledge of antibiotic use, which is higher than what we found in our study, which demonstrates that only 44.71% of the respondents had good knowledge. The low level of knowledge in our results is almost in line with a study that was done in Indonesia [16], in which only 52.98% of the respondents had good knowledge. In the Indonesian study, only 61.61% agreed that leftover antibiotics should not be used again, and only 37.0% knew that they should complete the full course of their antibiotics and that treatment should not be stopped if their illness has improved. Only 12.91% of the respondents agreed that antibiotics could not be used to treat infections due to viruses, and around 63.35% of the respondents think that antibiotics can be used to reduce fever. Inadequate knowledge was also reported in a Kuwait study [15], which showed that around 66.5% of the sample knew that antibiotics are effective against bacteria, and only 29.8% agreed that antibiotics are not used to treat viral illnesses. In the same study, around half (49%) of the respondents knew that unnecessary use of antibiotics can lead to resistance against them.

In the analysis of attitudes, more than half (59.5%) of the respondents had no problem ending an antibiotic course of treatment when their symptoms were improving, and this is concerning because not completing the course of treatment is one of the factors that can lead to the development of antibiotic resistance. Additionally, 48.7% of the respondents think that antibiotics can prevent any illness from becoming worse, and only 48.5% agree that natural sources may work better than antibiotics. Moreover, only 24.8% disagree that the use of antibiotics will speed up the recovery from a cold and cough. However, the majority (87.6%) trust the physicians' decisions to not prescribe antibiotics for their conditions. Attitudes results from the same previous Saudi study [13] showed that 92.55% of the total participants agreed to trust the physician's decision when deciding not to prescribe an antibiotic, 91.87% believed that antibiotics should not be given by doctors if not needed, and 91.33% disagreed with the accessibility of antibiotics without a prescription. On the contrary, about two-thirds (66%) did not believe that any illness could be prevented from worsening by the use of antibiotics. Additionally, 63.7% of the respondents believed that natural sources do not work better than antibiotics. The overall positive attitude score in this research was 76.80% of the respondents. Our findings were significantly lower. Only 12% of the participants in our study had good attitudes toward antibiotic use. The attitudes were no better in the Indonesian study [16]. Around 45% thought that the use of antibiotics could speed up recovery from a cold, and almost 30% were unsatisfied if they did not get antibiotics from their physician when they thought they needed them. Furthermore, approximately 35% of respondents had no problem purchasing antibiotics from pharmacies without a prescription, and half (50%) of the sample would discontinue their antibiotics as soon as they felt better. Additionally, a quarter (25%) stored leftover antibiotics for future use.

In the Kuwaiti study [15], only 57.6% showed a positive attitude towards completing the course of treatment, even if they felt better. 66.6% disagreed with buying antibiotics from a pharmacy without a prescription.

Respondents' practices towards antibiotic use showed that 26.1% get antibiotics from relatives without visiting a physician, and 42.1% expressed a negative practice of keeping antibiotic stock at home for future use. However, the majority (72.6%) agreed that antibiotics should not be accessed without a prescription, 77.9% disagreed with changing the physician for not prescribing antibiotics, and 73.9% disagreed with purchasing antibiotics from the pharmacy without a prescription. However, only 66.8% completed the course of antibiotics when they started it, and only 57.7% agreed to read the instructions on the label. Females showed better attitudes and practices compared to males. Higher income was associated with a better attitude, but it was insignificant in relation to practices. Higher levels of education had no significant effect on respondents' knowledge, attitudes, and practices toward antibiotics. Regarding practices, in the same previous Saudi study [13], most of the respondents (94.54%) disagreed with getting antibiotics from relatives regardless of the physician's prescription. 84.83% did not store antibiotics at home for future use. Additionally, the majority (92.64%) do not purchase antibiotics from the pharmacy without a prescription. Additionally, 93% of the respondents would not change their physicians if they did not get antibiotics. However, only 78% read the instructions on the label of the antibiotics. Good practices towards antibiotic use were found to be present in only 14.4% of the participants in the Saudi study [13]. A higher but inadequate level was found in our study, in which 39.12% of the participants were reported to have good practices. In the Indonesian study [16], around 40% of the respondents bought antibiotics from pharmacies without prescriptions, and 31% took leftover antibiotics to treat reoccurring symptoms. Regarding practices in the Kuwaiti study [15], around 64.7% of the respondents trusted physicians when they decided not to prescribe antibiotics, and around 30% preferred to keep leftover antibiotics for future use.

Although most of the respondents (26.1%) in our study do not purchase antibiotics without a medical prescription, many pharmacies in different regions of Saudi Arabia have been reported to be dispensing antibiotics to purchasers illegally. In 2001, a study was done in the eastern region of Saudi Arabia, in which the research team individually visited many community pharmacies complaining of urinary tract infection (UTI) symptoms. Only one pharmacy out of 88 refused to give medications without a prescription, and 80% of the given medications were antibiotics [9]. In 2011, the same approach to pharmacies was used in another study conducted in the Riyadh region. They found that out of 327 visited pharmacies, 244 (77%) illegally dispensed antibiotics [10].

The lack of sufficient knowledge about antibiotics and antimicrobial resistance leads to wrong beliefs that eventually manifest themselves as misuse and overuse of antibiotics. These incorrect practices, if not controlled, would accelerate the emergence of highly resistant organisms and their subsequent consequences. Several researchers have studied the topic of antibiotic awareness in Saudi Arabia. Recent research at the time of writing this paper was published in 2021 and studied the population of Jeddah city. Their work confirms our findings of poor KAP towards antibiotics and antimicrobial resistance in society [14].

Limitations

Efforts were made to get accurate, precise, and representative results. However, several limitations have been encountered during this work. First, the use of an online questionnaire to collect data has its own limitations. Second, the sample size for our study is small relative to the exact number needed for generalization. Therefore, all results are limited to our study participants only, and further research with a larger sample size is needed. In addition, most of the respondents to the questionnaire were females (75.6%), students (45.9%), with or studying for a bachelor's degree (70.7%). Third, respondents were not further categorized according to their cities or nationalities; rather, their responses were analyzed collectively, and the results were made for the western region entirely. These factors might have led to unintended biases in the results. However, our work gives us some understanding of the current situation and confirms the results of the previous studies that have been done on this subject globally and locally. Additional study in this field is required, with different approaches focusing on various sociodemographic traits and different regions of Saudi Arabia.

## Conclusions

The purpose of this study was to assess public knowledge, attitudes, and practices (KAP) toward antibiotic use and antimicrobial resistance. We concluded that most of our study participants had poor overall KAP. Our results highlight the knowledge gap regarding antibiotics and their appropriate usage. Thus, interventions need to be implemented to improve public KAP in this area. Some of the suggested interventions include educational health campaigns for the public that aim to promote awareness and enhance good practices. Moreover, enforcing more strict regulations to control the non-prescription dispensing of antibiotics is critical. However, further research on this topic is required to ensure that society is progressing in the right direction.

## References

[1] (2022). Antimicrobial resistance. https://www.who.int/news-room/fact-sheets/detail/antimicrobial-resistance.

[2] Department of Health and Social Care (2022). UK 5-year action plan for antimicrobial resistance 2019 to 2024. Tackling antimicrobial resistance 2019 to 2024: the UK's 5-year national action plan.

[3] Jamjoom GA, Azhar EI, Madani TA, Hindawi SI, Bakhsh HA, Damanhouri GA (2010). Genotype and antiretroviral drug resistance of human immunodeficiency virus-1 in Saudi Arabia. Saudi Med J.

[4] Al-Shahrani MS, Hakami MI, Younis MA, Fan HA, Jeraiby MA, Alraey Y (2021). Prevalence of primary anti-tuberculosis drug resistance at the tertiary center in Saudi Arabia and associated risk factors. Saudi Med J.

[5] Harbarth S, Balkhy HH, Goossens H (2015). Antimicrobial resistance: one world, one fight!. Antimicrob Resist Infect Control.

[6] Zowawi HM (2016). Antimicrobial resistance in Saudi Arabia. An urgent call for an immediate action. Saudi Med J.

[7] Al-Obeid S, Jabri L, Al-Agamy M, Al-Omari A, Shibl A (2015). Epidemiology of extensive drug resistant Acinetobacter baumannii (XDRAB) at Security Forces Hospital (SFH) in Kingdom of Saudi Arabia (KSA). J Chemother.

[8] Shibl AM, Memish ZA, Kambal AM, Ohaly YA, Ishaq A, Senok AC, Livermore DM (2014). National surveillance of antimicrobial resistance among Gram-positive bacteria in Saudi Arabia. J Chemother.

[9] Al-Ghamdi MS (2001). Empirical treatment of uncomplicated urinary tract infection by community pharmacist in the Eastern province of Saudi Arabia. Saudi Med J.

[10] Bin Abdulhak AA, Altannir MA, Almansor MA (2011). Non prescribed sale of antibiotics in Riyadh, Saudi Arabia: a cross sectional study. BMC Public Health.

[11] Leung E, Weil DE, Raviglione M, Nakatani H (2011). The WHO policy package to combat antimicrobial resistance. Bull World Health Organ.

[12] Benmerzouga I, Al-Zammay SA, Al-Shammari MM, Alsaif SA, Alhaidan TM, Aljofan M (2019). Practices of patients consuming antibiotics and knowledge about antibiotic resistance in Hail region - Saudi Arabia. Future Sci OA.

[13] Alnasser AH, Al-Tawfiq JA, Ahmed HA (2021). Public knowledge, attitude and practice towards antibiotics use and antimicrobial resistance in Saudi Arabia: a web-based cross-sectional survey. J Public Health Res.

[14] Zaidi SF, Baroom MW, Ibrahim Hanbashi A (2021). Cross-sectional survey among general population regarding knowledge and attitude toward antibiotic usage in western Saudi Arabia. Pharmacy (Basel).

[15] Awad AI, Aboud EA (2015). Knowledge, attitude and practice towards antibiotic use among the public in Kuwait. PLoS One.

[16] Karuniawati H, Hassali MA, Suryawati S, Ismail WI, Taufik T, Hossain MS (2021). Assessment of knowledge, attitude, and practice of antibiotic use among the population of Boyolali, Indonesia: a cross-sectional study. Int J Environ Res Public Health.

